# Building Engagement‐Capable Environments for Health System Transformation: Development and Early Implementation of a Capability Framework for Patient, Family and Caregiver Engagement in Ontario Health Teams

**DOI:** 10.1111/hex.70083

**Published:** 2024-11-03

**Authors:** Julia Abelson, Laura Tripp, Reham Abdelhalim, Lotje Hives, Betty‐Lou Kristy, Maureen Smith, Laura Tenhagen, Lindsay Wingham‐Smith, the Engagement Capable OHT Working Group

**Affiliations:** ^1^ Department of Health Research Methods, Evidence and Impact McMaster University Hamilton Ontario Canada; ^2^ Public and Patient Engagement Collaborative McMaster University Hamilton Ontario Canada; ^3^ Centre for Health Economics and Policy Analysis (CHEPA) McMaster University Hamilton Ontario Canada; ^4^ Burlington Ontario Health Team Burlington Ontario Canada; ^5^ Nipissing Wellness Ontario Health Team North Bay Ontario Canada; ^6^ Minister's Patient and Family Advisory Council Ministry of Health, Government of Ontario Toronto Ontario Canada; ^7^ Patient Partner Ontario Canada; ^8^ Sault Area Hospital Sault Ste. Marie Ontario Canada; ^9^ Mississauga Health Ontario Health Team Mississauga Ontario Canada

**Keywords:** engagement‐capable environments, framework development, patient engagement, patient involvement

## Abstract

**Introduction:**

Despite widespread calls to involve patients, families and caregivers (PFCs) as partners at all levels of health system planning and design, there is unevenness in how engagement efforts are supported across these settings. The concept of ‘engagement‐capable environments’ offers a way forward to uncover the key requirements for sustainable, high‐quality engagement, but more work is needed to identify the specific competencies required to create these environments. We addressed this gap by developing a capability framework for Ontario Health Teams (OHTs), a newly established structure for planning, designing, organizing and delivering care in Ontario, Canada.

**Methods:**

The framework was co‐developed by a Working Group of OHT staff and leaders, PFC partners, researchers and government personnel. Project activities occurred over four phases: (1) planning, (2) evidence review and surveying of intended users to identify key competencies, (3) framework design and (4) implementation.

**Results:**

An evidence review identified more than 90 potential competencies for this work. These results were contextualized and expanded through a survey of OHT stakeholders to brainstorm potential competencies, supports and enablers for engagement. Surveys were completed by 69 individuals; 689 knowledge and skill competency statements, 462 attitude and behaviour competency statements and 250 supports and enablers were brainstormed. The statements were analysed and organized into initial competency categories, which were reviewed, discussed and iteratively refined by Working Group members and through broader consultations with the OHT community. The final framework includes six competency domains and four support and enabler domains, each with sub‐domain elements, mapped across a three‐stage maturity model. The framework has been disseminated across OHTs, and its adoption and implementation are now requirements within OHT agreements.

**Conclusion:**

The framework combines a strong conceptual foundation with actionable elements informed by the literature and consultations with the intended users of the framework. Although developed for OHTs, the framework should be broadly applicable to other health system organizations seeking similar health system transformation goals.

**Patient Contribution:**

Patient, family and caregiver partners were involved at all stages and in all aspects of the work. As end users of the framework, their perspectives, knowledge and opinions were critical.

## 1 Introduction

The desire to improve the coordination, integration and responsiveness of health systems to meet the needs and preferences of patients and populations is a shared goal across many health systems. This is often combined with calls to centre patients, family members and caregivers (PFCs) in health system transformation efforts, typically through a combination of engagement, partnership and/or co‐design activities [1, 2, 3, 4, 5]. Despite these widespread calls, there has been a striking level of unevenness in how organizations approach the work of engaging patients and achieving the aims of more people‐centred health systems [6, 7, 8]. The significant decline in engagement work at the onset of the COVID‐19 pandemic reinforced its precarity and was associated with negative effects on performance outcomes [9, 10]. Yet, there are also examples of high‐performing organizations that put people with lived and living experiences at the centre of their work [10].

‘Engagement‐capable environments’ (ECEs) [11] offer a way forward to uncover the key requirements for sustainable, high‐quality engagement. The concept of ECEs was first introduced in quality improvement evaluation work [12] and more fully developed through case studies of organizations that are found to be uniquely able to create a culture of PFC engagement with the necessary infrastructure and support [1]. ECEs consist of *three inter‐related pillars*: creating space and capacity for patient partners, preparing and supporting staff and healthcare teams for engaging with patients and ensuring leadership support [13]. Recently, organizational culture has been identified as a key sustaining element for engagement capabilities [14]. Although the idea of creating ECEs has strong appeal, it requires considerable effort, integration into practice and a strategic commitment to resourcing to make these elements practical or ‘doable’. In particular, the knowledge, skills, attitudes, supports and enablers needed for those working in these environments are critically important for building ECEs, yet these have received comparatively little attention. Competency frameworks can be useful sources for identifying relevant knowledge, skills, attitudes and behaviours needed to support PFC engagement and partnering. At a broad level, competencies describe the essential knowledge, skills, attitudes and behaviours necessary in a specific field—in this case, PFC engagement and partnering. By identifying these elements, individuals and organizations can work towards building competencies that can be linked to different levels of capability and maturity in a specific area. Competency frameworks are commonly used in many professions, including healthcare [15, 16, 17].

Several efforts have been made to identify competencies across different constituency groups and sectors in the patient engagement field. At the direct care level, Bernabeo and Holmboe discuss the various competencies that patients and providers need to effectively engage in patient‐centred care, drawing on and adapting work by Towle and Godolphin [18, 19].

In their scoping review of patient‐oriented research competencies in health (PORCH), Frisch et al. set out to identify the core competencies needed by researchers, patients, healthcare providers and health system decision‐makers undertaking patient‐oriented research (POR) or POR‐related (or patient‐involved) research by systematically exploring peer‐reviewed and grey literature. Their results identified many competencies for researchers (e.g., engagement practices, communication and conflict management) and patients (e.g., research knowledge and skills, cultural competence and engagement practices), whereas fewer competencies were identified for healthcare providers or decision‐makers [20]. The patients active in research and dialogues for an improved generation of medicines (PARADIGM) multi‐stakeholder consortium outlined key competencies required for all stakeholders initiating patient engagement activities throughout the medicine development process, including five knowledge competencies, four skills competencies and three behavioural competencies [21].

Although competencies are important building blocks for ECEs, they are limited on their own without the organizational supports and structures required to enable them. For example, in addition to the patient and provider competencies outlined, Bernabeo and Holmboe also identified a set of ‘system competencies’ needed for supporting individual competencies [19]. Similarly, competencies are just one element of the PARADIGM capability model for patient engagement, which also includes processes, tools, systems and organizations [21]. Healthcare Excellence Canada's Engagement Capable Environments Organizational Self‐Assessment Tool supports competency development by guiding organizational leaders, staff, healthcare teams and patient partners through a self‐reflective exercise aimed at stimulating discussion about ‘where they are on their journey towards creating an engagement‐capable environment …’ [13, p. 6].

Progress has been made in articulating the pillars of ECEs and identifying the key competencies needed for those leading and working in these environments. However, these elements have not been integrated into a single framework, with a focus on placing patient engagement and people‐centred care at the centre of health system transformation efforts. We address this gap by developing a capability framework to support the implementation of patient engagement for people‐centred care within a newly established structure—Ontario Health Teams (OHTs)—that are aimed at achieving more coordinated, integrated and responsive health systems for patients and communities.

### 1.1 Setting and Context

This research was conducted in Ontario, Canada. In 2019, the provincial government introduced a major health system transformation designed to foster more integrated care through the establishment of OHTs. Comparable to accountable care organizations in the United States, OHTs are locally formed ‘groups of providers and organizations that, at maturity, will be accountable for delivering a full and coordinated continuum of care to an attributed population’ [22]. Currently, there are 58 OHTs across the province, serving every resident of Ontario. A core building block of the OHT model is the centring of PFC and community engagement and co‐design, which spans all OHT activities and governance. In this setting, PFCs refer to individuals with experience in the healthcare system themselves or as unpaid caregivers; these individuals can also be considered more broadly as community members. The requirements related to PFC engagement and partnership outlined in supporting OHT documentation have evolved and expanded since OHTs were first established. At the outset, OHTs were required to demonstrate their commitment to engagement and develop engagement and partnership strategies. Over time, greater priority has been given to ensuring that PFC partners are involved at key decision‐making tables at the executive governance level and that OHTs are co‐designing services and care models with PFC partners [23].

Although all OHTs have made progress towards centring PFC partners in their work, this has unfolded in different ways across the 58 OHTs, with different levels of supporting infrastructure. We used the concept of ECEs as an organizing framework to identify the key elements needed to build and support high‐quality PFC engagement, partnering and co‐design in the OHT context.

## 2 Methods

**Figure 1:**
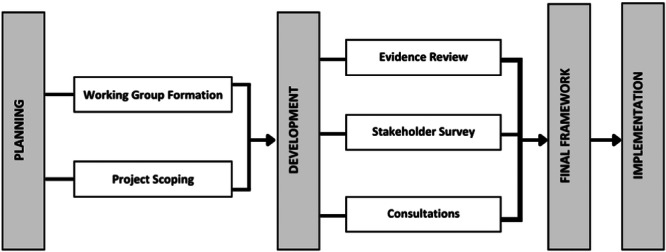
Framework development and implementation process.

The Public and Patient Engagement Collaborative, a research group at McMaster University and a member of the OHT Central Program of Supports, was tasked by the Government of Ontario's Ministry of Health to lead a small Working Group to develop competencies for supporting PFC engagement and partnership within OHTs. A multi‐phased process was undertaken to co‐develop the framework (Figure 1), to ensure that the final framework was relevant and acceptable to OHTs. Research ethics board approval was not sought as this was considered a quality improvement study.

### 2.1 Project Planning

#### 2.1.1 Working Group Formation

Working Group members were recruited with the goal of achieving diversity across roles and experiences (e.g., leadership, staff and PFC partners), as well as geographic representation from the six Ontario Health (OH) regions. The OHT Engagement Strategy [24] was used to facilitate this recruitment with support teams in each OH region soliciting requests for Working Group members to their constituent OHTs. One representative was selected from each region from those who put their names forward. Additional members were directly recruited by the research team to fill key gaps (e.g., additional PFC partner representation). Working Group members were involved in all stages of the project from the early scoping phases—where the aims and objectives for the work were confirmed—to the design and release of the final framework. PFC partners were offered an honorarium of $125 per meeting as compensation for their contributions. Compensation was not offered to Working Group members who were employed by their respective organizations.

#### 2.1.2 Project Scoping

Initial project scoping activities were undertaken to determine the key priorities and boundaries for the work, as well as the expectations of the sponsor (Government of Ontario) and key stakeholders (OHTs) to ensure that the final output met the needs of those who would be using the tool.

### 2.2 Framework Development

The framework was developed through three key phases: (1) evidence review, (2) stakeholder survey and (3) consultations. The Working Group was engaged throughout, providing guidance, insights and making key decisions.

#### 2.2.1 Evidence Review

The research team conducted a rapid review of the English‐language peer‐reviewed and grey literature [25, 26] to identify existing frameworks and examples of key competencies (e.g., skills, attitudes, behaviours and beliefs) required for supporting and implementing high‐quality PFC engagement. The review aimed to answer a key question driven by the project goals: ‘What are the competencies required for PFC engagement in health systems?’ The search aimed to identify existing competency frameworks in the area of health, broadly defined, along with educational programmes for PFC engagement. Three sources were searched for relevant competencies and frameworks: (1) OHT documents, (2) educational and training programmes and (3) the peer‐reviewed literature. Search terms for the peer‐reviewed literature included patient engagement, patient involvement, patient partnership, health, health systems, healthcare, competency, competencies, capability, capabilities, skill, knowledge, framework, tool and guide. Both Canadian and international frameworks were considered. All identified sources were reviewed for competencies that would be relevant to the OHT context, namely, competencies related to the skills, attitudes, behaviours and beliefs or structures that would support involvement in the high‐quality engagement of PFCs in health system programme/service design and policy, strategy and governance. Competencies focused on engagement at the direct care level were excluded. These were collated in an Excel database by three reviewers.

#### 2.2.2 Survey of OHTs

Informed by the evidence review and Working Group discussions, the framework development process was iterative and involved ongoing consultation and engagement with OHT and government partners to ensure that the final framework was relevant and tailored to the OHT context. Consultations began with a broad survey of OHT organizational leaders, including PFC partner leaders, clinicians and staff.

Using concept mapping methodology [27], a survey was developed for distribution in January 2023 to collect a list of potential competencies from OHT stakeholders for consideration for inclusion within the framework. The approach to survey development was discussed and agreed to in the first Working Group meeting. Survey questions were drafted by the research team for review and revision by Working Group members. The survey invited respondents to brainstorm competencies related to the knowledge, skills, attitudes and behaviours required for PFC partners and OHT staff, as well as enablers and supports that were needed at the organizational and government levels. Survey respondents were asked to complete the brainstorming prompts included in Table 1. Basic demographic questions were also included to allow for a description of the sample and to identify key gaps in the survey's reach.

**Table 1:** Survey questions.

Category	Prompts
Skills and Knowledge Competencies Brainstorming Prompt	The essential skills and knowledge that *OHT staff, leadership and clinicians* need to successfully engage with patients, families and caregiver partners in OHTs are…
	The essential skills and knowledge that *patient, family and caregiver* partners need to successfully engage with Ontario Health Teams are…
Attitudes and Behaviour Competencies Brainstorming Prompt	The attitudes and behaviours that *OHT staff, leadership and clinicians* need to successfully engage with patients, families and caregiver partners in OHTs are…
	The attitudes and behaviours that *patient*, *family and caregiver partners* need to successfully engage with Ontario Health Teams are…
Structures and Supports Brainstorming Prompt	For Ontario Health Teams to effectively engage with patient, family and caregiver partners, the structures and supports that need to be in place at an organization/system level include…
Demographics	Role within OHT
	Age group
	Gender identity
	Cultural background
	Level of experience with PFC engagement
	Region (location)

A multi‐pronged approach to recruitment was used to ensure wide distribution of the survey to relevant stakeholders: (i) a survey flyer was sent to regional teams within Ontario Health with a request to share the survey across the OHT system; (ii) Working Group members promoted the survey within their networks and OHTs; (iii) the survey link was shared with members of the Community of Practice (CoP) on PFC Engagement and Partnering in OHTs and with members of the Minister's Patient and Family Advisory Council (MPFAC) and (iv) the survey was promoted on Twitter/X by the lead author (J.A.).

Data were analysed using NVivo and summarized in Excel. Brainstormed competency statements related to knowledge, skills, attitudes and behaviours were sorted into broad thematic categories and sub‐categories by groups (PFC partners and staff). Brainstormed statements for structures and supports were also sorted into broad thematic categories. During this process, duplicate items were removed.

#### 2.2.3 Consultations

Further consultations were conducted with key informants from OHTs to provide additional insights into the proposed competencies and to further contextualize the results. Emphasis was placed on reaching out to key informants with experience and expertise in equity, diversity and inclusion, as a key focus of this work was to ensure that the framework facilitated equity‐centred engagement.

### 2.3 Final Framework Development

Results from the evidence review, survey and consultations were reviewed and discussed with the Working Group over three meetings. At the first meeting, initial survey results and analysis were shared to review the overall approach and identify natural groupings of items. In the second meeting, proposed competency domains and sub‐domains were shared, with decisions made about the structure and organization of competency categories. Additional feedback was obtained from Working Group members via email between meetings. These were then mapped onto a three‐level maturity model developed with the Working Group to demonstrate the process of building capability in this area.

To support capacity building, the Working Group aimed to include a list of resources and professional learning opportunities for each competency domain. A review of existing supports and professional learning opportunities for PFC engagement within OHTs and beyond was conducted, focusing on Ontario‐developed supports where available. These were then mapped to the identified competencies, structures and enablers for inclusion in the final framework.

### 2.4 Implementation

An implementation planning process was conducted in consultation with the Working Group, the Ministry of Health and Ontario Health partners. Key areas of focus included knowledge translation—sharing the framework as widely as possible across the OHT community; policy supports—understanding how the framework would be implemented within the OHT policy context; and implementation supports—developing additional tools to support the framework's implementation.

## 3 Results

The Working Group met four times over a 4‐month period to develop the framework, with additional interactions by e‐mail and in small groups between formal meetings. The Working Group was co‐chaired by the lead of the Public and Patient Engagement Collaborative (J.A.) and the Chair of the MPFAC (B‐L.K.) and included engagement leads, PFC partners, OHT leadership and Ministry of Health representatives.

### 3.1 Phase 1: Project Planning

#### 3.1.1 Project Scoping

A number of project scoping activities were undertaken to ensure that the project would be relevant to, and meet the needs of, individuals working in and with OHTs. Initial meetings were held with the Ministry of Health partners to scope the work and the workplan, as well as with the OHT PFC Engagement and Partnering CoP, an online community that hosts monthly meetings to support OHT staff, partners and others in their engagement efforts. These initial conversations provided background information and context to the need for the framework and some important considerations. The CoP members highlighted, for example, their concerns surrounding the language of ‘competencies’ given its dual meaning in a medical context around an individual's decision‐making capacity and how this may be triggering for some, especially for those partners with lived experience within the mental health sector.

The first Working Group meeting focused on sharing initial findings from the evidence review and finalizing the scope of the project based on the initial feedback received from the groups consulted. The group agreed that the key aims of the project were to (1) develop a competency framework for PFC engagement and partnering in OHTs linked to a maturity model and (2) identify training/support opportunities for the achievement of these competencies. The group agreed that the focus of the framework would be on engagement at the programme and service design and policy, strategy and governance levels and not at the direct care level [28]. The importance of bringing an equity lens and approach to this work was highlighted by Working Group members and framed subsequent phases. It was agreed that not only must there be an equity focus within the competencies, but the individuals consulted on the development of the framework should include individuals with expertise in equity‐centred engagement. Additional scoping conversations were held in terms of how the framework would be used—if it would serve as a guidance document for OHTs to refer to and consider in their work or if it would serve as a policy document that OHTs would be required to follow and implement. At the time of the project scoping, it was understood that the framework would serve as general guidance for OHTs.

The terminology to be used within the framework was discussed in detail, given the concerns raised regarding the term ‘competency’. The Working Group identified the importance of using language that is easy to understand across various groups. Concerns about the term ‘competency’ extended beyond its dual meanings in healthcare settings; there was also the idea that competency is often seen as something you either have or do not. However, the most important issue is whether and how you apply that competency to the work. Several other terms were considered, including tools, skill sets, capabilities and enablers (for individuals and organizations).

#### 3.1.2 Evidence Review

The ECE literature provided a foundation for understanding the concepts of building organizations that are well‐positioned for engagement [1, 11, 13]. A number of competency frameworks and relevant literature from related fields were also identified including patient‐centred and caregiver‐centred care [19, 29, 30, 31, 32], health system improvement [8], engagement in drug development [21, 33] and engagement in patient‐oriented research [20]. Documents related to OHTs were also reviewed to identify relevant competencies, including the *Patient, Family and Caregiver Declaration of Values for Ontario* [34] and relevant government policies [22, 23]. Frameworks, policies and publications were reviewed to identify unique competencies of relevance to this work. More than 90 were identified through the review.

The results of the evidence review were shared with Working Group members. Although many of the competencies identified were of interest and potential relevance, there was an identified need to determine what competencies were of specific relevance to OHTs through further consultation.

### 3.2 Phase 2: Survey of OHTs

Surveys were completed by 69 individuals across 66 surveys (one survey was completed by four individuals as a group). Of these, 49 surveys (52 individuals) included completed demographic information. Most respondents were PFC partners (56%) or OHT staff (23%), and most indicated that they were extremely or moderately experienced with PFC engagement (77%). Surveys were completed by individuals working within each of the OHT regions except for one (Toronto) with variation across the regions in terms of the number of completed surveys (Table 2). Among those who provided demographic information, respondents were primarily women (77%) and reported a European cultural background (62%).

**Table 2:** Demographic characteristics of survey respondents.

Demographic characteristics	*N* (%)
Role^a^	
Patient, family and caregiver partner	56% (29)
OHT leadership	15% (8)
OHT staff	23% (12)
OHT partner	15% (8)
Age	
19–34 years	17% (9)
35–54 years	31% (16)
55 or older	42% (22)
No response	10% (5)
Gender identity	
Man	17% (9)
Woman	77% (40)
Non‐binary person	0% (0)
Prefer not to answer	6% (3)
Cultural background^a^	
African	2% (1)
European	62% (32)
South Asian	6% (3)
South East Asian	2% (1)
First Nations or Indigenous	4% (2)
Prefer not to answer/no response	19% (10)
Other	10% (5)
Level of experience with PFC engagement	
Slightly or somewhat experienced	23% (12)
Moderately or extremely experienced	77% (40)
OH region	
North East	23% (12)
North West	2% (1)
East	11% (6)
Central	29% (15)
Toronto	0% (0)
West	29% (15)
Provincial level	2% (1)
No response	4% (2)

a Categories were not mutually exclusive; respondents could select more than one.

In response to the survey prompt questions, respondents brainstormed 689 statements regarding knowledge and skill competencies required (326 for PFC partners and 363 for OHT staff) and 462 statements regarding attitude and behaviour competencies required (213 for PFC partners and 249 for OHT staff). A further 250 statements related to structures and supports were identified (Table 3).

**Table 3:** Brainstorming survey results.

Competency type	Number of brainstormed statements
Patient, family and caregiver partners	
Knowledge and skills	326
Attitudes and behaviours	213
OHT staff	
Knowledge and skills	363
Attitudes and behaviours	249
General	
Structures and supports	250

Brainstormed statements were reviewed by the research team. Duplicate statements were removed, and statements were grouped into themes across the competency types (knowledge, skills, attitudes and behaviours) for OHT staff and PFC partners. Structures and supports were grouped into an enabler sub‐group. When reviewing the brainstormed statements, there was poor conceptual distinction both between the competency categories (knowledge/skills, attitudes/behaviours) and between the role categories (PFC partners and staff). Many of the same or similar competencies were identified in both categories, either by the same respondent or across respondents. Preliminary results were shared with the Working Group over three meetings, which provided guidance on further refinement of the competency groupings during meetings and by email between meetings.

**Figure 2:**
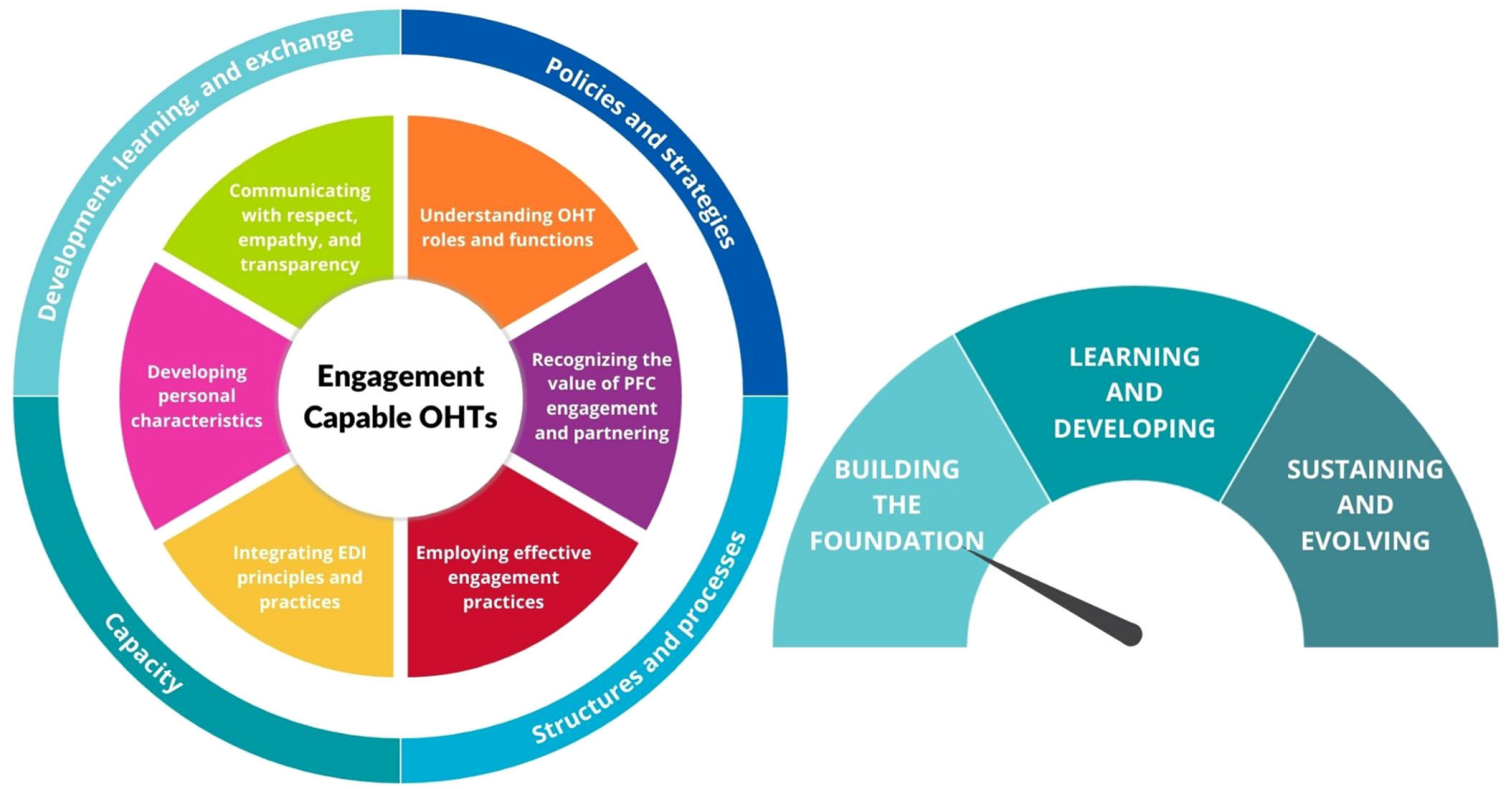
Engagement‐capable Ontario Health Team Framework.

Two key decisions were made to address the high degree of overlap identified across the competency categories and target groups, which informed subsequent analysis and framework development:

1. The original competency categories (knowledge, skills, attitudes and behaviours) were replaced with overarching domains and sub‐domains across competency types (Figure 2), while maintaining specific wording related to competency types.
2. Competency statements were combined across target groups (no longer separated for PFC partners and staff); any tailoring required for specific groups was to be inserted in the wording of a particular competency.

### 3.3 Phase 3: Consultations

Efforts were undertaken to consult more broadly on the proposed framework to address the lack of diversity in the survey sample (as described in Section 2). Key insights from these consultations identified the need to expand the language used to recognize OHTs' roles related to health and social services; include greater sensitivity to the challenges faced by certain populations in building knowledge and trust in local health and social systems (e.g., individuals without access to the Ontario Health Insurance Plan [OHIP], newcomers) and how this influences capacity and wiliness to engage and the importance of considering accountability to ensure that the framework is implemented within OHTs. Consultations also highlighted the importance of considering a range of engagement approaches that not only consider the preferred options for organizations but also for different communities and populations. Modifications were made to various elements of the framework based on the feedback received through these consultations.

### 3.4 Resource Development

The final framework includes three key elements viewed as integral to building engagement‐capable OHTs. These include *six competency domains* and *four supports and enabler domains* (Figure 2), each described in detail with numerous examples and sub‐points (Tables 4 and 5). The framework includes links to *relevant capacity‐building resources* for each of the domains. A *maturity model* is presented highlighting how each of these domains looks in practice across three maturity stages. The maturity stages acknowledge that building an engagement‐capable environment takes time and is an ongoing process. Key expectations are outlined for each of the three stages of maturity: (1) building the foundation, (2) learning and developing and (3) sustaining and evolving.

The framework was released in June 2023 and is hosted on the Public and Patient Engagement Collaborative's website in both web‐based and printable PDF formats available in English and French: https://ppe.mcmaster.ca/engagement-capable-oht-framework/.

#### 3.4.1 Competency Domains

Competency domains identify the key capabilities that are required by OHT staff and leadership and OHT PFC partners for engagement to be successful within the OHT. These include a number of knowledge and skills domains, as well as values and characteristics that need to be fostered and developed within individuals in the OHT for this work to be successful. These domains and selected sub‐domain elements are outlined in Table 4.

**Table 4:** Competency domains and selected sub‐domain elements.

Domain	Selected sub‐domain elements
1. Understanding OHT roles and functions in relation to health and social service systems	Understand the structure, purpose and role of an OHT, including the roles of all members including PFC partners Understand the basic structure and funding arrangements of health and social service systems provincially and at their local OHT level Recognize that some groups may have limited understanding of these systems beyond their own experiences and may need additional support to build this knowledge
2. Recognizing the value of PFC engagement and partnering	Understand the centrality of PFC partners' perspectives to patient‐centred health systems Demonstrate commitment and passion for PFC engagement Appreciate, recognize and celebrate the contributions and successes of PFC partners
3. Employing effective PFC partnership and engagement practices	Understand the meaning of partnership and engagement in the OHT context and the different forms this can take Understand the principles and approaches required to foster safe and respectful engagement and partnering Understand the key requirements for effective partnership and engagement Understand the role of evaluating PFC engagement and partnering as part of a learning and improvement culture
4. Integrating equity, diversity and inclusion (EDI) principles and practices into all engagement and partnering work	Understand the key concepts of EDI and how they relate to each other Be aware of and practice the principles of reconciliation and Indigenous cultural safety Understand how EDI principles may be applied differently across different communities and populations Understand accessibility requirements to ensure all can actively participate in engagement activities
5. Developing personal characteristics to enhance meaningful and authentic engagement	Be aware of the personal characteristics that contribute to meaningful and authentic engagement Approach engagement activities with compassion and empathy Demonstrate awareness of personal/unconscious biases and actively work towards reducing/managing them
6. Communicating with respect, empathy and transparency	Use active listening skills in all interactions to promote mutual understanding Use different language and communication styles to ensure cultural sensitivity and accessibility Effectively share lived experiences to inform system change in a trauma‐informed way

#### 3.4.2 Support and Enabler Domains

Support and enabler domains provide the infrastructure that OHT leadership, staff and PFC partners need to work together in an environment that fosters respectful and effective engagement. In many cases, these are not under the direct control of OHTs and need to be developed in consultation with their funders (Ministry of Health, Ontario Health). Working Group members and those with whom we consulted highlighted the importance of having supports and enablers in place, as these are critical to the success of the overall functioning of PFC engagement within the OHTs. The four domains and selected sub‐domain elements for each are highlighted in Table 5.

**Table 5:** Support and enabler domains and selected sub‐domain elements.

Domain	Selected sub‐domain elements
1. Policies and strategies	OHT policies and strategies demonstrate a clear commitment to PFC engagement and its centrality to all OHT activities Policies and procedures to support PFC engagement and partnering are in place with clearly identified roles for OHT staff and PFC partners Communication strategies to support PFC engagement and partnership are in place
2. Structures and processes	Engagement structures with clear goals and operational plans (e.g., PFAC) are in place including the inclusion of PFC partners on governance tables Supports for PFC partner roles and activities are developed and shared including recruitment and onboarding strategies and role descriptions Procedures for directly supporting PFC partners are clearly described and transparently communicated including compensation guidelines and tools and supports to remove barriers to participation
3. Capacity	Strong OHT leadership with training in PFC engagement Dedicated staff member with specific knowledge and training in PFC engagement Dedicated budget for PFC engagement and detailed funding guidelines for compensating PFC partners Measurement and evaluation capacity to support learning and improvement around PFC engagement and partnering strategies
4. Development, learning and exchange	Ongoing development and learning opportunities for staff and PFC partners are available and funded through the OHT Ongoing opportunities for learning and exchange Tailored development, learning and exchange support for specific groups (e.g., Northern OHTs)

### 3.5 Implementation

The framework has been shared extensively through the OHT community through webinars and direct communication. Two webinars were held to introduce OHTs to the framework and highlight the role of the framework in collaborative decision‐making. Additional presentations and discussions were held with the PFC Engagement and Partnering CoP and with a provincial working group of support partner organizations focused on PFC engagement to support the framework's adoption and implementation.

In May 2024, the requirement to adopt and implement the framework was included in OHTs' agreements with their funding agency, with requirements to complete “an assessment of the current state and an action plan outlining how the OHT will advance to a minimum of Level 2: Learning and Developing” [35]. The inclusion of the framework as part of multi‐year OHT agreements addressed many of the concerns raised both by Working Group members and during consultations regarding how to ensure OHTs would see the adoption of this framework as a priority in a context where there are many requirements and areas of focus. Several implementation supports have been introduced or repurposed to promote the uptake of the framework as OHTs work towards creating engagement capability environments. A self‐assessment tool has been developed to assist OHTs with understanding the current state of their PFC engagement and partnering work and to plan for further development in this area [36]. The framework is being used as the key organizing structure for a CoP focused on building knowledge, skills and capacity for high‐performing engagement, with monthly meetings dedicated to supporting the implementation of framework elements. CoP activities will be enhanced by coaching activities tailored to priority areas identified by OHTs, and a dedicated training offering has been introduced to build expertise and capacity in PFC engagement across the OHT community.

## 4 Discussion

The principles of ECEs have been around for at least a decade and are slowly taking root in health system organizations. Competency frameworks for patient engagement are also beginning to proliferate in specific areas (direct care, drug development and patient‐oriented research). Our framework builds on these parallel areas of activity in an integrated way by establishing the key competencies and supports required to create and sustain ECEs for health system transformation. Our work adds to the patient engagement competency literature by focusing on competencies at the organizational and governance level. It extends ECE thinking by using it as an organizing framework for developing a comprehensive resource that includes individual competencies, organizational supports and enablers needed along with links for building capacity in each area across defined maturity stages.

Ideas about how to support good engagement practice have been known for some time but organizational commitment and implementation challenges have been persistent barriers to achieving measurable progress on this front. Our model of using rigorous methods while working in close collaboration with the intended implementers of this resource–organizational leaders, engagement specialists and PFC partners in OHTs– was viewed as critical to maximizing the potential for the final resource (*the framework*) to be viewed as both evidence‐informed *and* usable by our target implementers. This involved representation from each of these constituencies in a small, time‐limited Working Group complemented by surveys and targeted consultations with key constituencies through all stages of the framework development process. This level of involvement influenced our work in significant ways. For example, our initial emphasis on developing a competency framework was re‐shaped by two key inputs: (i) staff emphasized that without attention to organizational supports and enablers, competencies alone would fall short of achieving the aims of ECEs; and (ii) members of our PFC partner community raised concerns about potential confusion with the legal meaning of competency in relation to patient decision‐making. Although we have retained the use of the term ‘competency’ in the framework, this insight was instrumental in ensuring that our language was clear to reduce confusion and that it did not become the sole focus of our work. Another substantive contribution was the idea of not segmenting the competencies by group (e.g., leadership, staff and PFC partners) to reinforce the idea of teams and organizations working together in all areas. This addresses concerns previously documented that the work of PFC engagement is sometimes left to PFC partners and that other members of the organization including leadership team members also require skills in this area. The flexible, iterative and continuous approach to engaging with Working Group members and key constituencies throughout the framework development process, along with our commitment to ongoing communication, was key to ensuring that each contributor felt heard, understood and valued throughout the process. The very enablers and competencies to which we refer within this framework were made visible in our approach to engagement and were key to the success of this work.

## 5 Limitations

Our work is not without limitations. Although we sought to reach a wide range of OHT stakeholders through our consultations, our brainstorming survey was completed by predominately older women with a European background. The lack of diversity in this sample was identified as a concern by the Working Group given the interest in ensuring that the framework appropriately and adequately addressed concepts of equity, diversity and inclusion and was relevant to a large range of users. We attempted to mitigate this limitation by consulting with groups focused on equity and inclusion within OHTs and the Ontario Health system more broadly but acknowledge that this does not replace hearing from individuals directly. This is an ongoing issue for patient engagement in Canada. Previous work has identified that there is a lack of diversity among patient partners in Canada, with the majority being older, retired, women of a European background [6]. This highlights the importance for OHTs, and others working in the engagement space, to engage with PFC partners in flexible and responsive ways to encourage broader representation among patient partners. OHTs are working to address these challenges through a range of strategies well recognized within the field of community engagement, including outreach to existing community groups and the hiring of community ambassadors to help facilitate this outreach. The emphasis on these principles as one of the competency domains in our framework will hopefully help to address these challenges over time.

Furthermore, survey respondents identified that they had a high level of experience with engagement. Although it was not unexpected to reach individuals with a high level of experience with engagement given our target populations, individuals with less experience in engagement may have identified different capabilities of interest.

## 6 Conclusion

The framework developed and presented in this article combines a strong conceptual foundation with actionable elements informed by a synthesis of published and grey literature and extensive input from key health system partners who are the intended users of the framework. Although the framework was developed for a particular constituency of health system organizations in a single jurisdiction in Canada, the competency areas and support and enabler categories should be broadly applicable to other organisations pursuing similar health system transformation goals.

## Author Contributions

**Julia Abelson:** conceptualization, formal analysis, funding acquisition, investigation, methodology, project administration, supervision, visualization, writing–original draft. **Laura Tripp:** data curation, formal analysis, investigation, methodology, project administration, visualization, writing–original draft. **Reham Abdelhalim:** methodology, visualization, writing–review and editing. **Lotje Hives:** methodology, visualization, writing–review and editing. **Betty‐Lou Kristy:** methodology, visualization, writing–review and editing. **Maureen Smith:** methodology, visualization, writing–review and editing. **Laura Tenhagen:** methodology, visualization, writing–review and editing. **Lindsay Wingham‐Smith:** methodology, visualization, writing–review and editing.

## Ethics Statement

As this was considered a quality improvement study, no ethics approval was required.

## Conflicts of Interest

The authors declare no conflicts of interest.

## Data Availability

The data that support the findings of this study are available from the corresponding author upon reasonable request.
